# Health Care Personnel Exposures to Subsequently Laboratory-Confirmed Monkeypox Patients — Colorado, 2022

**DOI:** 10.15585/mmwr.mm7138e2

**Published:** 2022-09-23

**Authors:** Kristen E. Marshall, Marlee Barton, Janell Nichols, Marie A. de Perio, David T. Kuhar, Emily Spence-Davizon, Meghan Barnes, Rachel K. Herlihy, Christopher A. Czaja, Colorado Healthcare Personnel Monitoring Team, Colorado Healthcare Personnel Monitoring Team, Theo Abbey, Alyssa Beck, Jennifer Bernal, Tori Burket, Connor Carrillo, Mary Casey, Karen Daily, Catherine Emanuel, Sonakshee Havis, Jillian Jaskunas, Mike Kacka, Ella Keenan, Grace Nelson, Eileen Tran, Leslee Warren, Saher Yunus

**Affiliations:** ^1^Colorado Department of Public Health and Environment; ^2^Career Epidemiology Field Officer Program, CDC; ^3^CDC Monkeypox Emergency Response Team.

The risk for monkeypox transmission to health care personnel (HCP) caring for symptomatic patients is thought to be low but has not been thoroughly assessed in the context of the current global outbreak (*1*). Monkeypox typically spreads through close physical (often skin-to-skin) contact with lesions or scabs, body fluids, or respiratory secretions of a person with an active monkeypox infection. CDC currently recommends that HCP wear a gown, gloves, eye protection, and an N95 (or higher-level) respirator while caring for patients with suspected or confirmed monkeypox to protect themselves from infection^†^ (*1*,*2*). The Colorado Department of Public Health and Environment (CDPHE) evaluated HCP exposures and personal protective equipment (PPE) use in health care settings during care of patients who subsequently received a diagnosis of *Orthopoxvirus* infection (presumptive monkeypox determined by a polymerase chain reaction [PCR] DNA assay) or monkeypox (real-time PCR assay and genetic sequencing performed by CDC). During May 1–July 31, 2022, a total of 313 HCP interacted with patients with subsequently diagnosed monkeypox infections while wearing various combinations of PPE; 23% wore all recommended PPE during their exposures. Twenty-eight percent of exposed HCP were considered to have had high- or intermediate-risk exposures and were therefore eligible to receive postexposure prophylaxis (PEP) with the JYNNEOS vaccine^§^; among those, 48% (12% of all exposed HCP) received the vaccine. PPE use varied by facility type: HCP in sexually transmitted infection (STI) clinics and community health centers reported the highest adherence to recommended PPE use, and primary and urgent care settings reported the lowest adherence. No HCP developed a monkeypox infection during the 21 days after exposure. These results suggest that the risk for transmission of monkeypox in health care settings is low. Infection prevention training is important in all health care settings, and these findings can guide future updates to PPE recommendations and risk classification in health care settings.

CDPHE collected data on clinical and nonclinical HCP exposed by treating, being within 6 feet of, or handling linens from a patient who subsequently received a diagnosis of monkeypox in health care settings during May 1–July 31, 2022. CDPHE interviewed patients with monkeypox and reviewed medical records to ascertain whether lesions were present during health care exposures. HCP who had cared for out-of-state patients or for patients who were lost to follow-up were excluded from the analysis. Exposure details, including types of PPE worn, types of interaction, and amount of time spent with the patient were collected for each HCP, and risk levels were assigned using the CDC HCP risk assessment criteria at the time (low or uncertain, intermediate, or high). HCP with high- or intermediate-risk exposures were offered JYNNEOS PEP vaccination and were actively monitored for symptoms for 21 days after the exposure.^¶^ HCP with low-risk exposures were asked to self-monitor for symptoms for 21 days.** HCP who experienced symptoms were asked to notify CDPHE immediately, were excluded from work until symptoms resolved, and received *Orthopoxvirus* testing if rash or lesions occurred. All HCP included in the analysis completed the 21-day monitoring period. In addition, facilities reported all exposure, PPE, and exposure risk data to CDPHE. PEP administration data were obtained from reporting facilities and through the Colorado Immunization Information System. These data were summarized and stratified by facility type and job title. Analyses were completed using R statistical software (version 2021.09.2; The R Foundation). This activity was reviewed by CDC and was conducted consistent with applicable federal law and CDC policy.^††^

During May 1–July 31, 2022, a total of 313 HCP were exposed to 55 patients with monkeypox, including 20 high-risk, 67 intermediate-risk, and 226 low- or uncertain-risk exposures (Table). Seven HCP had exposure during aerosol-generating procedures; three of whom wore an N95 respirator during their exposure. Overall, 273 (87%) exposures to patients with monkeypox rash or lesions occurred, and 161 (59%) included direct contact with the patient’s skin or lesions (gloves were worn in 125 exposures, were not worn in 30 exposures, and use of gloves was unknown for six exposures). Twenty-six (8%) exposed HCP reported handling linens; 23 (88%) of whom were wearing gloves. Approximately two thirds of encounters with monkeypox patients (215; 69%) lasted 5–30 minutes. Only one health care worker was exposed for >3 hours; this health care worker wore an N95 respirator and all other recommended PPE for the duration of the exposure.

**TABLE:** Health care personnel exposures to monkeypox patients, by facility type — Colorado, May 1–July 31, 2022

Exposure	No. (subsection %)			
	Total	Community health and STI	Hospitals and EDs	Urgent or primary care
**Total**	**313 (100)**	**25 (100)**	**175 (100)**	**113 (100)**
Risk classification*				
High^†^	**20 (6)**	2 (8)	8 (5)	10 (9)
Intermediate	**67 (21)**	4 (16)	33 (19)	30 (27)
Low or uncertain	**226 (72)**	19 (76)	134 (77)	73 (65)
**Aerosol-generating procedure^§^** **performed**	**7 (2)**	0 (—)	7 (4)	0 (—)
N95 respirator use during aerosol-generating procedure	**3 (43)**	NA	3 (43)	NA
**Lesions present during patient encounter**	**273 (87)**	25 (100)	159 (91)	89 (79)
**Touched patient when lesions were present**	**161 (59)**	12 (48)	102 (64)	47 (53)
Glove use	**125 (78)**	9 (75)	85 (83)	31 (66)
No glove use	**30 (19)**	3 (25)	12 (12)	15 (32)
Unknown glove use	**6 (4)**	0 (—)	5 (5)	1 (2)
**Handled linens**	**26 (8)**	0 (—)	23 (13)	3 (3)
Glove use	**23 (88)**	NA	22 (96)	1 (33)
No glove use	**3 (12)**	NA	1 (4)	2 (67)
Unknown glove use	**0 (—)**	NA	0 (—)	0 (—)
**Duration of exposure**				
<5 mins	**22 (7)**	1 (4)	12 (7)	9 (8)
5–30 mins	**215 (69)**	14 (56)	106 (61)	95 (84)
>30 mins–3 hrs	**53 (17)**	6 (24)	42 (24)	5 (4)
>3 hrs^¶^	**1 (0)**	0 (—)	1 (1)	0 (—)

**Abbreviations:** ED = emergency department; NA = not applicable; STI = sexually transmitted infection.

* Risk classification determined by CDC Health Care Worker Exposure Criteria. http://web.archive.org/web/20220615195256/https:/www.cdc.gov/poxvirus/monkeypox/clinicians/monitoring.html

^†^ One needlestick injury occurred in a community health and STI facility during phlebotomy and was considered a “high-risk” exposure; this health care worker received postexposure prophylaxis vaccination and did not develop monkeypox.

^§^ Aerosol-generating procedures included intubation and endoscopy.

^¶^ The health care worker with >3 hours of exposure wore an N95 respirator during their entire period of potential exposure.

Among the 313 exposed HCP, 118 (38%) reported wearing an N95 respirator while treating or interacting with monkeypox patients. N95 respirator use by HCP varied among health care settings: 64% of exposures of HCP in community health and STI clinics, 50% in hospitals and emergency departments (EDs), and 12% in primary and urgent care settings occurred while the health care worker was wearing an N95 respirator (Figure). Among the 72 (23%) HCP who wore all recommended PPE while treating monkeypox patients, all were classified as low or uncertain risk. Adherence to all recommended PPE ranged from 4% in primary and urgent care settings to 48% in community health and STI clinics. Clinical staff members reported higher PPE use than did nonclinical staff members, with providers and nurses reporting the highest compliance with recommendations (Supplementary Table, https://stacks.cdc.gov/view/cdc/121197).

**FIGURE:**
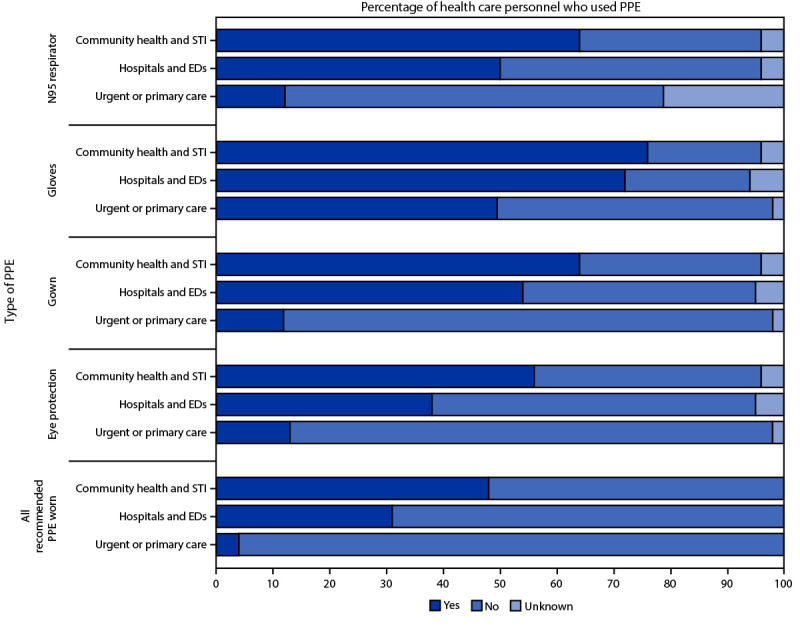
Personal protective equipment use by health care personnel* exposed to patients with monkeypox, by facility type — Colorado, May 1–July 31, 2022 **Abbreviations:** ED = emergency department; PPE = personal protective equipment; STI = sexually transmitted infection. * Number of health care personnel by facility type: community health and STI (25), hospitals and EDs (175), and urgent or primary care (113).

HCP with intermediate- and high-risk exposures (87; 28%) were eligible to receive PEP with JYNNEOS vaccine (Table). Among eligible HCP, 37 (43%) received PEP, including 10 (50%) with high-risk exposures and 27 (40%) with intermediate-risk exposures. Seven of the 313 exposed HCP experienced symptoms during their 21-day monitoring period; three had rash or lesions, and four had other nonspecific symptoms. Two of the three HCP with rash or lesions were tested for *Orthopoxvirus*; both PCR test results were negative, and the third health care worker had an alternative diagnosis for their rash (medication reaction).

## Discussion

In the United States, data suggest that widespread community transmission of monkeypox has occurred in the context of sexual or close intimate contact (*3*). In Colorado, monkeypox transmission did not occur to 313 HCP with varying levels of exposure to patients with monkeypox during patient care or through contaminated materials. These findings are consistent with literature review from previous U.S. outbreaks (*1*) and internationally imported cases (*4*,*5*), with one case report of transmission to a health care worker after contact with contaminated patient linens in the United Kingdom during a previous outbreak (*6*), and one case of transmission to a health care worker in the United States during the current outbreak (*7*). Most HCP exposures in this analysis (72%) were classified as low or uncertain risk; only seven HCP (2%) were exposed during an aerosol-generating procedure. These data are consistent with evidence that occupationally acquired monkeypox is unlikely to occur when adhering to recommended infection prevention and control precautions.

Only 23% of exposed HCP wore all recommended PPE. Although mask use was common, likely because of current COVID-19 source control recommendations, only 38% of HCP wore N95 respirators, and 64%, 40%, and 31% wore gloves, gowns, and eye protection, respectively. These low percentages might have been due to lack of awareness of 1) a patient’s symptoms before entering their care area, 2) community transmission, 3) monkeypox PPE recommendations, or 4) monkeypox signs and symptoms or atypical presentation (*3*).

These data suggest that opportunities exist to improve awareness and training among frontline HCP who are most likely to see patients with monkeypox, so that they can take steps to protect themselves from exposure. The need for increased awareness and preparation was most apparent in primary care and urgent care settings where adherence to recommended PPE use was lowest. STI clinics became referral centers in Colorado, which might explain the higher PPE adherence in these settings. PPE use varied by job type as well, with more clinical providers and nursing staff members typically wearing recommended PPE than nonclinical staff members.

The findings in this report are subject to at least four limitations. First, higher compliance in community health and STI clinics might not be generalizable nationwide because these sites were used as referral centers in Colorado; these clinics were often informed of patients with suspected monkeypox before the patient arrived and were aware of current PPE and infection control recommendations. Second, PEP vaccination data were limited and might have been underreported. The Colorado Immunization Information System verification process was limited because of potential typographical errors in HCP names or dates of birth, as well as whether a health care worker consented to their information being entered into the system. Third, data on exposures to contaminated materials were incomplete, limiting the ability to draw conclusions regarding this potential route of transmission. Finally, information about whether patients had covered lesions or worn facemasks during their health care visits was unavailable.

This study illustrated that the risk for HCP acquiring monkeypox after exposure to patients with monkeypox was very low despite incomplete adherence to recommended PPE, especially among primary and urgent care settings, and receipt of PEP by fewer than one half of eligible exposed HCP. Despite these gaps, no HCP in Colorado developed monkeypox during their 21-day monitoring period. These results underscore the importance of public health outreach to better understand the circumstances of HCP exposures so that prevention, infection prevention education, and training of HCP can be improved, especially in primary care and urgent care settings. In addition, these data might support future updates to PPE use recommendations and exposure risk classification in health care settings.

**Summary**

**What is already known about this topic?**

Although risk for monkeypox transmission to health care personnel (HCP) is thought to be low, CDC recommends that HCP wear personal protective equipment (PPE) consisting of gown, gloves, eye protection, and an N95 (or higher-level) respirator while caring for patients with suspected or confirmed monkeypox.

**What is added by this report?**

Among 313 Colorado HCP exposed to patients with monkeypox, recommended PPE use and receipt of postexposure prophylaxis vaccination was low. HCP were assessed for risk and actively monitored for 21 days when indicated; none acquired monkeypox.

**What are the implications for public health practice?**

The risk for acquiring monkeypox among U.S. HCP after exposure to patients with monkeypox is very low. HCP in all health care settings can benefit from public health outreach regarding infection prevention education and training.

## Footnotes

* These authors contributed equally to this report.

†
https://www.cdc.gov/poxvirus/monkeypox/clinicians/infection-control-healthcare.html

§ https://www.fda.gov/vaccines-blood-biologics/jynneos

¶ http://web.archive.org/web/20220615195256/https:/www.cdc.gov/poxvirus/monkeypox/clinicians/monitoring.html

** All exposed HCP received the recommended 21 days of monitoring after exposure, including those excluded from the analysis.

†† 45 C.F.R. part 46, 21 C.F.R. part 56; 42 U.S.C. Sect. 241(d); 5 U.S.C. Sect. 552a; 44 U.S.C. Sect. 3501 et seq.
